# Chronic Disease and Disability in the United States: Trends, Inequities, and Imperatives for Policy Reform

**DOI:** 10.1111/1468-0009.70109

**Published:** 2026-08-14

**Authors:** ALI H. MOKDAD, LAURA DWYER‐LINDGREN, CHRISTOPHER J.L. MURRAY

**Affiliations:** ^1^ Institute for Health Metrics and Evaluation University of Washington

## Abstract

**Policy Points**

- ***Position Universal, High‐Quality Education as a Foundational Public Health Strategy***. Long‐term health and life expectancy in the United States are shaped more by educational attainment than by any other single factor. Disparities in access to early childhood, K‐12, and higher education perpetuate cycles of chronic disease, economic hardship, and shortened lifespan, particularly in marginalized communities. Policymakers must recognize education as a critical public health intervention and commit to providing universal access to high‐quality education at every stage of life. This transformation requires shifting the focus from mere per‐pupil spending toward the national scaling of high‐impact instructional models, such as intensive, high‐dosage tutoring and adaptive learning. Leveraging generative AI to provide personalized, real‐time instruction tailored to individual student needs is essential to bridge the achievement gap at scale. Furthermore, achieving true educational equity requires decoupling school quality from local property taxes, integrating health literacy and nutrition into curricula, and ensuring higher education remains affordable and accessible for all.
- ***Transform the Food and Built Environment to Advance National Metabolic Resilience***. The structure of America's food system and built environment directly fuels the escalating burden of obesity, diabetes, and related chronic diseases. Federal and state policies must fundamentally shift agricultural subsidies away from calorie‐dense, nutrient‐poor commodities toward specialty crops and fresh produce. To ensure these healthy choices are the default for every household, the federal government must finalize and enforce mandatory, interpretive front‐of‐package warning labels for ultra‐processed foods. This shift, designed to move beyond the passive Nutrition Facts panel, will provide at‐a‐glance transparency and incentivize industrywide reformulation. Furthermore, metabolic health must be integrated into the K‐12 experience through mandatory daily high‐intensity physical activity to improve cardiorespiratory fitness and cognitive performance and minimally processed school meals. Finally, investments in active transportation infrastructure, including pedestrian greenways and safe routes to school, are essential to encourage movement as a default, closing the “convenience gap” that disproportionately burdens low‐income and rural Americans.
- ***Normalize Chronic Obesity Management***. Acknowledging that no nation has successfully reversed the obesity epidemic through behavioral advice alone, the United States must treat obesity as a chronic biological condition rather than a temporary lifestyle lapse. It is imperative that public and private payers guarantee universal, affordable access to evidence‐based treatments, including next‐generation pharmacotherapies (such as GLP‐1 receptor agonists). These clinical strategies must be coupled with a modernized, real‐time health data infrastructure to monitor outcomes and ensure equitable access at the neighborhood level.

**Context:**

Over the past three decades, mortality from leading causes such as cardiovascular disease and cancer has declined in the United States, but the burden of chronic, disabling conditions, including musculoskeletal disorders, mental and substance use disorders, and obesity, has grown or stagnated, widening the gap between total years lived and years lived in good health. These shifts are accompanied by persistent and widening inequities in longevity by geography, race/ethnicity, and socioeconomic status.

**Methods:**

This analysis draws on the 2023 Global Burden of Disease Study, which estimates mortality, disability, and risk factor burden for 375 diseases and 88 risk factors nationally and at the state level, combined with findings from the US Health Disparities project. The latter uses small‐area estimation to evaluate county‐level life expectancy and mortality across five racial/ethnic groups and four educational strata, and the “Ten Americas” framework, which stratifies the population by race/ethnicity, geography, and socioeconomic context.

**Findings:**

In 2023, US life expectancy reached 78.5 years and healthy life expectancy 64.5 years, both still below prepandemic 2019 levels, with a persistent 13‐14‐year gap between life expectancy and healthy life expectancy. Ischemic heart disease remained the leading cause of death, but drug use disorders rose from the 26th to the leading cause of disability, a 562% increase since 1990, while Alzheimer disease and musculoskeletal disorders also climbed in rank. High systolic blood pressure remained the top mortality risk factor despite a large decline, while high body mass index and high fasting plasma glucose burdens grew substantially. State‐level disparities widened, with West Virginia and Mississippi trailing Hawaii by wide and increasing margins. Life expectancy gaps by race/ethnicity, education, and place were large and growing, reaching 20.4 years across the “Ten Americas” by 2021, with county‐level life expectancy spanning more than 27 years nationally.

**Conclusions:**

US health trends reflect a shift from fatal to disabling disease, driven substantially by the obesity/metabolic and substance use epidemics, layered onto deep and widening structural inequities by race/ethnicity, place, and education. Reversing these trends requires a multidimensional policy agenda spanning universal, high‐quality education; reform of the food and built environment; normalized clinical management of obesity; expanded, integrated substance use and mental health treatment; and modernized, real‐time public health data infrastructure.

Over the past three decades, the united states has experienced profound shifts in the burden of chronic disease and disability among adults. Although mortality from leading causes such as cardiovascular disease and cancer has declined, the prevalence and subsequent burden of chronic conditions, including musculoskeletal disorders, mental and substance use disorders, obesity, and chronic respiratory disease, have increased or stagnated.^1^, ^2^, ^3^ This has widened the gap between total years lived and years lived in good health. These trends are paralleled by stark and growing inequities in longevity, as highlighted by our recent analyses partitioning the US population into “Ten Americas.”^4^ The result is a segmented national health landscape in which geography, race, ethnicity, and socioeconomic status increasingly determine not only the risk of death but also the burden of disability.^5^, ^6^, ^7^, ^8^, ^9^, ^10^, ^11^, ^12^, ^13^

This divergence over the last three decades reveals that survival gains have decoupled from gains in disability‐free life.^1^, ^2^, ^14^, ^15^, ^16^, ^17^, ^18^, ^19^, ^20^, ^21^, ^22^ As Americans live longer, they are increasingly doing so with more disabilities. The COVID‐19 pandemic both exposed and exacerbated mental disorders. Our recent study shows that depending on one's race, ethnicity, geographic location, and economic context, one's life expectancy can differ by more than 20 years.^4^ Understanding the drivers, distribution, and consequences of chronic disability is thus essential for effective health policy. This paper synthesizes national evidence on adult chronic disease trends and offers actionable strategies to address the growing and unequal burden of poor health in the United States. We used the 2023 Global Burden of Disease Study (GBD 2023)^23^, ^24^, ^25^, ^26^ and our US Health Disparities^6^, ^7^, ^8^, ^9^, ^10^, ^11^ project to present the current burden. All methodology details, sources of data, and results were published previously.^6^, ^7^, ^8^, ^9^, ^10^, ^11^, ^12^, ^13^, ^23^, ^24^, ^25^, ^26^, ^27^, ^28^, ^29^, ^30^

## Global Burden of Disease 2023 Methods

The GBD 2023 study quantifies the national and state‐level burden of 375 diseases and 88 risk factors.^23^, ^24^, ^25^, ^26^ This framework uses the Cause of Death Ensemble model (CODEm) and Bayesian meta‐regression (DisMod‐MR 2.1) to ensure robust comparisons across time and geography. We use evidence‐based algorithms to redistribute “garbage codes,” ill‐defined, intermediate, or implausible causes of death (e.g., heart failure, senility, unspecified cancer), back to the most probable underlying cause. This methodology was fully described previously, including how the uncertainty intervals of the estimates are computed.^24^ In the United States, the GBD 2023 applies garbage code redistribution to 12.6% of deaths; this ranged from 8.4% in South Dakota to 21.3% in Alabama. This process improves public health data accuracy and mortality comparison. The number and age‐standardized rate of cause‐specific deaths were estimated using CODEm to assess the out‐of‐sample predictive validity of different statistical models and combine results into mortality estimates. Life expectancy at birth and for each age group was calculated using age‐specific mortality rates and standard demographic methods. Years of life lost (YLLs) were computed as the product of the number of deaths and the standard life expectancy at each age.

For nonfatal disease and injury modeling, cause‐specific incidence and prevalence were modeled using DisMod‐MR 2.1. This Bayesian tool was utilized for all but four causes, type 1 diabetes, depression, anxiety, and autism spectrum disorder, which used DisMod‐AT. To estimate years lived with disability (YLDs), GBD 2023 used a microsimulation approach, calculating the prevalence of sequelae and multiplying these by disability weights derived from community surveys. This included a comorbidity correction to account for the co‐occurrence of nonfatal causes within individuals. Disability‐adjusted life years (DALYs) were computed by summing YLDs and YLLs. Healthy life expectancy (HALE), measuring mean years spent in good health, was estimated using YLDs per capita and age‐specific mortality rates. To ensure comparability across disparate data sources, we applied rigorous cross‐walking and bias‐adjustment methods to standardize nonstandard disease definitions and diagnostic measurements to a preferred reference case definition. This process accounts for variations in clinical assays, diagnostic instruments, and survey methodologies, ensuring that the resulting YLD estimates reflect true epidemiological changes rather than artifacts of data collection.

Risk factor modeling followed a comparative risk assessment framework. We estimated the relative risk of health outcomes as a function of exposure using burden of proof meta‐regression. Summary exposure values and theoretical minimum risk exposure levels were used to compute the population attributable fraction for each risk‐outcome pair. Finally, our US Health Disparities project integrated small‐area estimation models to evaluate life expectancy and mortality at the county level for five mutually exclusive racial/ethnic groups: American Indian and Alaska Native (AIAN), Asian or Pacific Islander (Asian), Black, Latino, and White, alongside educational levels. This subnational work relies on National Vital Statistics System death records and American Community Survey population data, utilizing specific misclassification corrections to ensure racial identity is accurately represented.

Data sources and processing in this study are available in the GBD 2023 Sources Tool (https://ghdx.healthdata.org/gbd‐2023/sources).

## Life Expectancy and Healthy Life Expectancy

### National Trends

In 2023, US life expectancy was 78.5 years (95% UI, 78.48‐78.51), a 3‐year increase from 75.5 years in 1990. Despite a postpandemic rebound, it remains below the 2019 level of 78.9. In 2023, female life expectancy (81.1 years) significantly exceeded that of males (75.9 years). National HALE reached 64.5 years in 2023, up from 63.5 in 1990 but still below the 2019 level of 65.4.

### State‐Level Variation

Between 1990 and 2023, life expectancy at birth increased in most states and the District of Columbia. The lowest life expectancy was in Mississippi (73.9 years) and the highest in Hawaii (81.1 years). The largest gains occurred in the District of Columbia, New York, New Jersey, and California, while West Virginia (−0.3), New Mexico (−0.01), and Oklahoma (+0.02) saw little to no improvement.

HALE patterns mirrored these inequities. In 2023, West Virginia recorded the lowest HALE (59.9 years) and Hawaii the highest (67.5 years). Between 2019 and 2023, the pandemic reduced HALE in nearly every state. The gap between life expectancy and HALE remained stable at 13‐14 years, indicating no significant reduction in the duration of life lived in poor health.

## Mortality

### National Trends

In 2023, ischemic heart disease (IHD) remained the leading cause of death in the United States, with an age‐standardized mortality rate (ASMR) of 75.37 deaths per 100,000 (95% UI, 66.66‐81.16). This represents a 58.7% decrease compared to 1990, though it retained its top rank. Next, Alzheimer disease was the second leading cause, with a rate of 29.98 (7.41‐73.18), essentially unchanged from 1990 (−0.1%) but rising in rank from fourth in 1990 to second in 2023. Stroke held the third position, with a rate of 29.93 (25.88‐32.97), a significant 35.7% reduction since 1990. Chronic obstructive pulmonary disease (COPD) ranked fourth, with 28.89 (25.46‐31.73), up 8.9% since 1990, moving slightly up from fifth to fourth. The fifth leading cause was drug use disorders, with a rate of 25.97 (20.79‐31.24), which is a staggering 1,186% increase compared to 1990, and a dramatic jump in rank from 50th in 1990 to fifth in 2023. Lung cancer dropped from the second rank to sixth and had a 48.7% reduction. These patterns highlight major public health shifts: while traditional chronic diseases like IHD and stroke have decreased substantially, Alzheimer disease burden has become more prominent, and an explosive rise in drug use disorder deaths has brought it into the top five, reflecting the ongoing opioid crisis.

### State‐Level Variation

In 1990, District of Columbia (977 per 100,000) and Louisiana (790) had the highest ASMR, while Hawaii (537) had the lowest. By 2023, West Virginia had the highest ASMR (737), and Hawaii remained the lowest (432). The absolute gap between the highest‐ and lowest‐performing states decreased from 440 deaths per 100,000 in 1990 to 305 in 2023. But it would have increased if District of Columbia, an outlier, was excluded, indicating an increase in disparities. During the pandemic, all states experienced mortality spikes, with West Virginia, Mississippi, and New Mexico seeing the largest increases. Male mortality remains higher across all states, with Mississippi reporting the highest 2023 male ASMR (891.8). Female mortality gaps widened significantly over the study period, with the interquartile range across states increasing.

Causes of age‐standardized deaths varied by states, but IHD was the leading cause in all states in 2023. However, there was large variation in the second cause with COPD in 17 states, stroke in ten states, Alzheimer disease in 13 states, drug use disorders in nine states and District of Columbia, and hypertensive heart disease in Oklahoma. IHD was the leading cause for males and for females, except in Hawaii, where Alzheimer disease was the leading cause for females (Table 1).

**Table 1:** Top Ten Causes of Age‐Standardized Deaths: United States, 2023

Rank	Cause	Deaths per 100,000	95% UI (Lower‐Upper)	Percentage Change Since 1990, %	1990 Rank → 2023 Rank
1	Ischemic heart disease	75.37	66.66‐81.16	−58.7	1 → 1
2	Alzheimer disease and other dementias	29.98	7.41‐73.18	−0.1	4 → 2
3	Stroke	29.93	25.88‐32.97	−35.7	3 → 3
4	Chronic obstructive pulmonary disease	28.89	25.46‐31.73	+8.9	5 → 4
5	Drug use disorders	25.97	20.79‐31.24	+1,186	50 → 5
6	Tracheal, bronchus, and lung cancer	25.66	23.61‐27.41	−48.7	2 → 6
7	Chronic kidney disease	23.59	19.97‐26.29	+189.8	19 → 7
8	Cirrhosis and other chronic liver diseases	13.73	12.75‐14.73	+17.3	12 → 8
9	Hypertensive heart disease	13.71	12.18‐14.94	+93.2	21 → 9
10	Diabetes mellitus	13.09	11.51‐14.74	−13.6	10 → 10

## Disability

### National Trends

In 2023, drug use disorders became the leading cause of age‐standardized disease burden (DALYs) in the United States, skyrocketing to 2,312.5 DALYs per 100,000 (95% UI, 1,933.5‐2,717.5)—a dramatic 562% increase since 1990, when it ranked only 26th. This sharp rise reflects the ongoing opioid epidemic and the broad impact of substance use on health. IHD, while still imposing a major burden (1,579.9 DALYs; UI, 1,453.5‐1,669.7), has dropped to second (from first) as its burden fell by more than half (–56%), highlighting advances in prevention and treatment. Low back pain is now the third leading cause, with 1,161.9 DALYs (UI, 834.6‐1,523.6), relatively stable (–8%) but rising in rank from fourth. Falls have decreased in overall burden (–20%, now 1,045.4 DALYs; UI, 798.2‐1,343.9), slipping from second to fourth. Rounding out the top five, other musculoskeletal disorders increased notably (+41% to 961.3 DALYs; UI, 691.2‐1,250.3) and climbed from tenth to fifth place. The US burden of disease has shifted from primarily cardiovascular‐ and injury‐related causes to a profile where chronic pain, musculoskeletal conditions, and especially substance use disorders predominate. This represents both victories in traditional public health (declining heart disease) and new challenges, particularly the opioid crisis and the management of chronic, disabling conditions.

### State‐Level Variation

Age‐standardized DALYs rates fell in 39 states between 1990 and 2023 but rose in 12, concentrated in Appalachia, the Southwest, and the Ozarks; West Virginia, Oklahoma, New Mexico, Kentucky, and Tennessee had the largest increases. West Virginia (35,802 per 100,000) and Hawaii (24,118) represent the highest and lowest burdens. Drug use disorders were the leading age‐standardized cause for DALYs for both sexes combined in all states except for Arkansas, Mississippi, Texas, Iowa, Nebraska, North Dakota, and South Dakota. However, it was the leading cause in all states for ages 20‐54, while IHD was the leading cause for ages 55 or older. For ages 10‐19, depressive disorders were the leading cause in 21 states, anxiety disorders in 23 states, interpersonal violence in two states and District of Columbia, road injuries in three states, and musculoskeletal in one state (Table 2).

**Table 2:** Top Ten Causes of Age‐Standardized DALYs: United States, 2023

2023 Rank	Cause	DALYs per 100,000	95% UI (Lower‐Upper)	Percentage Change Since 1990, %	1990 Rank → 2023 Rank
1	Drug use disorders	2,312.5	1,933.5‐2,717.5	+562	26 → 1
2	Ischemic heart disease	1,579.9	1,453.5‐1,669.7	−56	1 → 2
3	Low back pain	1,161.9	834.6‐1,523.6	−8	4 → 3
4	Falls	1,045.4	798.2‐1,343.9	−20	2 → 4
5	Other musculoskeletal disorders	961.3	691.2‐1,250.3	+41	10 → 5
6	Diabetes mellitus	892.6	717.3‐1,100.0	+29	9 → 6
7	Anxiety disorders	820.0	542.0‐1,194.4	+25	11 → 7
8	Depressive disorders	817.6	568.8‐1,137.1	+42	15 → 8
9	Road injuries	813.5	740.7‐883.4	−37	3 → 9
10	Stroke	727.4	648.9‐797.9	−29	7 → 10

## Risk Factors

The risk factor landscape shaping US mortality and disability in 2023 reveals both the enduring impact of public health achievements and the emergence of formidable new threats, particularly those driving chronic disease. While some traditional risks have declined, others, most notably high body mass index (BMI), high fasting plasma glucose (FPG), and drug use, have surged, fundamentally altering patterns of health loss across the population.

### National Trends

High systolic blood pressure remains the leading risk factor for mortality in the United States, though its age‐standardized impact has fallen dramatically by 44.5% since 1990 (from 132.9 to 73.7 deaths per 100,000). This marked reduction highlights the cumulative benefits of decades of hypertension awareness, screening, and treatment. However, these successes stand in sharp contrast to the rising tide of high BMI and high FPG, which have become leading risk factors in the nation's chronic disease burden. Deaths attributable to high BMI rose by 11.8% (to 55.8 per 100,000), and the associated disability burden (DALYs) increased by 24.3% (to 2,267 per 100,000) since 1990, making high BMI the second leading risk factor for both premature death and disability. High FPG, a marker for diabetes and related metabolic dysfunction, was the third leading risk for DALYs, with an increase of 15.1% and a decrease in ASMR of 6.6%.

Alongside these metabolic risks, the United States faces a rapidly worsening crisis of substance use. ASMR and age‐standardized DALYs have surged: since 1990, DALYs attributable to drug use have increased by 340%, now surpassing smoking and high blood pressure as the nation's leading cause of disability. This trend is especially pronounced among young and middle‐aged adults, reflecting the devastating impact of the opioid epidemic and broader polysubstance use. Smoking, while still a major contributor, has seen dramatic reductions in both mortality (down nearly 50%) and DALYs (down 48%), demonstrating the effectiveness of sustained control policies. Nevertheless, smoking remains a top five risk, particularly among males and in certain geographic regions.

### State‐Level Variation

Geographic disparities in risk factor burden are pronounced and widening. From 1990 to 2023, every state experienced a decline in ASMR attributable to all risk factors, but the magnitude of improvement was highly uneven. Hawaii consistently reported the lowest rates (217.8 deaths per 100,000 in 2023), while West Virginia remained the highest (432.6), reflecting the persistence of deep structural inequities. The difference in DALY rates between the lowest and highest states (excluding the District of Columbia) grew from 5,398 to 8,416 per 100,000 over the period. The District of Columbia, New York, New Jersey, and California achieved the largest reductions in risk‐attributable DALYs, whereas West Virginia, New Mexico, Oklahoma, and Kentucky saw the largest increases, indicating a worsening burden of chronic disease and injury.

High blood pressure was the leading risk for ASMR in all states for both sexes combined, except for Kentucky where smoking was the leading risk. For females, high blood pressure was the leading risk factor in all states, but for males, smoking was the leading risk in six states, drug use in New Mexico, and high blood pressure in the rest of the states and District of Columbia. There was variation by age in risk factors for deaths with drug use as the leading cause for both sexes combined for ages 20‐54 and for females and for males, except in South Dakota, where high alcohol use was the leading risk for ASMR. For age‐standardized DALYs, drug use was the leading cause for both sexes combined for all ages in 36 states and the District of Columbia and high BMI in 14 states (Table 3 and Table 4).

**Table 3:** Top Ten Risk Factors for Age‐Standardized Deaths: United States, 2023

Rank	Risk Factor	Deaths per 100,000	95% UI (Lower‐Upper)	Percentage Change Since 1990, %	1990 Rank → 2023 Rank
1	High systolic blood pressure	73.70	59.90‐87.46	−44.5	1 → 1
2	High body mass index	55.85	32.18‐76.80	+11.8	6 → 2
3	High fasting plasma glucose	55.27	47.56‐65.10	−6.6	4 → 3
4	Smoking	54.94	44.94‐66.85	−48.7	2 → 4
5	Kidney dysfunction	39.74	32.93‐45.75	−9.3	7 → 5
6	Drug use	33.95	28.96‐39.47	+329.3	25 → 6
7	High LDL cholesterol	27.27	17.72‐39.37	−59.5	3 → 7
8	High alcohol use	21.03	17.28‐26.71	+13.9	12 → 8
9	Low temperature	14.21	12.40‐15.49	−40.2	10 → 9
10	Lead exposure	13.58	10.62‐16.74	−60.6	8 → 10

**Table 4:** Top Ten Risk Factors for Age‐Standardized DALYs: United States, 2023

Rank	Risk Factor	DALYs per 100,000	95% UI (Lower‐Upper)	Percentage Change Since 1990, %	1990 Rank → 2023 Rank
1	Drug use	2,592.97	2,220.24‐2,997.95	+340.5	12 → 1
2	High body mass index	2,267.18	1,121.26‐3,286.41	+24.3	3 → 2
3	High fasting plasma glucose	1,759.33	1,490.09‐2,070.24	+15.1	5 → 3
4	Smoking	1,726.81	1,327.05‐2,158.94	−48.0	1 → 4
5	High systolic blood pressure	1,537.90	1,250.38‐1,786.63	−39.5	2 → 5
6	High alcohol use	1,099.64	924.42‐1,330.74	+3.0	7 → 6
7	Kidney dysfunction	895.40	787.98‐1,014.50	+3.7	9 → 7
8	Sexual violence against children	874.35	295.97‐1,561.43	+60.6	13 → 8
9	High LDL cholesterol	709.14	484.98‐975.82	−55.2	4 → 9
10	Low birth weight and short gestation	594.77	544.97‐647.24	−36.7	8 → 10

## Place‐Based, Racial and Ethnic, and Educational Disparities

### National Disparities in Life Expectancy by Race and Ethnicity

National gains in life expectancy over the past two decades have been deeply uneven across racial/ethnic groups in the United States. Between 2000 and 2019, the Latino (+2.7 years), Asian (+2.9 years), and Black (+3.9 years) populations saw meaningful increases, whereas gains among White Americans were more modest (+1.7 years). The AIAN population, however, saw no net improvement in life expectancy over this period. Consequently, the gap between the highest and lowest life expectancy groups widened substantially, reaching 12.5 years in 2019 (comparing the Asian and AIAN populations). Importantly, these national patterns obscure even greater diversity at the local level, with the magnitude and direction of disparities shifting dramatically by place.

### County‐Level Variation in Life Expectancy by Race and Ethnicity

Beneath national averages, life expectancy varied profoundly across US counties, both within and among racial/ethnic groups. In 2019, county‐level life expectancy ranged from 64.5 to 91.7 years for the total population, a difference of more than 27 years. For White populations, the county span was 67.4‐91.9 years; Black populations, 66.5‐88.8 years; AIAN, 58.6‐93.3 years; Asian, 78.5‐92.7 years; and Latino, 71.3‐94.9 years. The direction and size of race/ethnicity gaps depended heavily on geography: in some counties, the gaps were similar to what was observed nationally, while in others, the gaps were reversed or far wider than national averages. For example, while life expectancy for Black Americans was lower than for White Americans in 87% of counties, there was a range from a deficit of over 15 years to an advantage of almost 8 years. These striking local differences highlight the powerful influence of place‐based social, economic, and environmental conditions on longevity.

### County‐Level Variation in Healthy Life Expectancy by Race and Ethnicity

Disparities were also stark for HALE. In 2019, county‐level HALE ranged from 55.1 to 76.2 years for the total population, a 21.2‐year difference. AIAN populations faced the greatest disadvantage: the ten counties with the lowest HALE for AIAN populations were also the lowest for any group‐county combination, with Neshoba County, Mississippi, reporting an AIAN HALE of just 50.1 years. For Asian populations, HALE ranged from 64.9 to 77.3 years; Latino populations, from 60.3 to 77.0 years; White populations, from 55.0 to 77.3 years; and Black populations, from 56.4 to 79.1 years. Notably, local patterns sometimes diverged from national trends, e.g., in 44 counties, Latino populations had higher HALE than Asian populations, and in 139 counties, Black populations had higher HALE than White populations. These findings illustrate how both longevity and health quality are shaped by the intersection of race/ethnicity, place, and local context.

### Educational Disparities in Life Expectancy

Disparities by educational attainment have also grown sharply. From 2000 to 2019, college graduates lived 8.2‐10.7 years longer than those without a high school diploma. The educational gradient was consistent: at age 25, those with a college degree had higher life expectancy than those with some college education (by up to 2 years), who in turn outlived high school graduates (by 4‐5 years), and so on. County‐level variation was extreme—even among those with the same education. In 2019, life expectancy for people without a high school diploma ranged from 57.9 to 90.1 years depending on county, a difference of over 32 years. In more than 90% of counties, the gap between the most educated and the least widened over time, showing that education and place interact powerfully to determine health outcomes.

## Disease‐Specific Outcomes: National and County‐Level Disparities by Race and Ethnicity

Examining disease‐specific outcomes reveals profound disparities by race/ethnicity at the national level as well as striking heterogeneity across counties. Nationally, ASMRs for major chronic and acute conditions show consistently higher burdens among historically marginalized groups.

For diabetes in 2019, the ASMR was highest among the AIAN population at 35.6 per 100,000, followed by the Black (31.9), Latino (19.7), White (17.6), and Asian (12.6) populations. The highest county‐level diabetes mortality rateswere in counties in Mississippi and South Dakota. For Black populations, some counties in the Mississippi Delta had diabetes mortality rates over 100 per 100,000, far exceeding both White and Latino populations in those same areas.

Mortality rates for liver cancer in 2019 also showed persistent racial/ethnic disparities. AIAN, Black, Latino, and Asian populations all experienced higher burdens than White populations, though the patterns varied by region. For example, Asian populations saw the highest liver cancer mortality rates in certain Kansas, Iowa, Minnesota, South Dakota, and Texas counties, while AIAN populations had the highest rates in certain California, Maine, Minnesota, Montana, and South Dakota counties.

Lung cancer mortality showed a different pattern. Nationally, the highest ASMR in 2019 was among the AIAN population (49.3 per 100,000), followed by the Black (47.8), White (45.8), Asian (24.2), and Latino (20.6) populations. County‐level variation was also substantial: the five highest ASMR in 2019 for Black populations were in Louisiana (two counties), Ohio, Pennsylvania, and Virginia; for White populations, in Kentucky (four counties) and Virginia.

Pregnancy‐related mortality increased among all racial/ethnic groups between 2000 and 2019. Nationally, the highest rates were observed among Black (3.7 deaths per 100,000 females aged 15 to 44 years) and AIAN (3.5) women, followed by White (1.4), Latina (1.3), and Asian (1.0) women. The fastest relative increases were seen among White women, with median county‐level increases exceeding 200% since 2000. Some counties with large AIAN or Black populations experienced rates that were severalfold higher than the national average, consistent with compounding effects of structural barriers and local health care access.

## “Ten Americas” Framework: Synthesizing Place, Race and Ethnicity, and Socioeconomic Context

Given the immense complexity and volume of county‐level data, we applied the Ten Americas framework to group the population into ten distinct subpopulations defined by race/ethnicity, geography, metropolitan status, residential segregation, and socioeconomic conditions. Life expectancy gaps across these Americas were large and growing, from 12.6 years in 2000 to 20.4 years in 2021, with a sharp acceleration during the COVID‐19 pandemic. America's most advantaged group, Asian individuals (America 1), had life expectancy 83.1 years in 2000, while the least advantaged, AIAN individuals in the West (America 10), saw life expectancy decline to 63.6 years by 2021. The three Black Americas (Americas 6, 7, 9) experienced substantial gains before 2020, overtaking some White Americas, while America 10 saw declines even before the pandemic. The ordering and gaps between Americas changed over time, reflecting dynamic interactions among segregation, rurality, concentrated poverty, and race/ethnicity.

## Individual‐Level Human Development Index: Patterns and Change

To further integrate the multidimensional nature of well‐being, we adapted the Human Development Index (HDI) to the individual level, combining years of education, household consumption, and expected lifespan from 2008 to 2021. Mean HDI increased gradually for most groups until 2019, then declined in 2020 due to pandemic‐related drops in lifespan. Profound inequalities marked the HDI distribution: in the lowest decile, about half of AIAN males and 40% of Black males were represented, alongside 23% of AIAN females and 21% of Latino males. However, White males, due to population size, composed the largest number in the lowest decile (27%, 5.87 million). Age patterns were striking: among ages 25‐44, 66% of AIAN males and 46% of Black males were in the lowest HDI decile, while among the oldest adults, women predominated. People in the lowest HDI decile were disproportionately concentrated in the South, Appalachia, and the Rust Belt.

## Current Landscape of US Health

Our research reveals a persistent and troubling trend: the overall health status of the US population is not keeping up with advances in peer nations, regardless of the metric used. Despite a modest gain in life expectancy and HALE over three decades, these increases are minimal relative to advances seen in countries like Australia, the United Kingdom, France, and Spain. Even the healthiest US states now lag behind many high‐income and some middle‐income countries in HALE. Perhaps most concerning is the widening gulf between the best‐ and worst‐performing states: the difference in life expectancy, HALE, and DALYs between states such as Hawaii and Minnesota on one end and Mississippi and West Virginia on the other has grown, not narrowed, over time.

The persistent disadvantage faced by AIAN and Black populations across counties highlights that health inequities are baked into the structural and environmental fabric of the nation. As our HDI analysis shows, when educational attainment and household resources are considered alongside longevity, the erosion of human potential is most acute among young males. This lost potential is not merely a clinical failure but a systemic one.

Taken together, these analyses demonstrate that US health disparities are multidimensional, dynamic, and deeply entrenched. Building on our previous publications,^3^ this work highlights how geography, race/ethnicity, and socioeconomic context interact to produce dramatic inequities that have not only persisted but accelerated during the COVID‐19 pandemic.

These results underscore a significant and ongoing failure of health policy to address the nation's poor performance relative to other high‐income countries. The fundamental question is no longer whether a gap exists, but what must be done to reverse this steady decline. Addressing these disparities requires moving beyond individual or disease‐focused interventions toward place‐based, structural strategies that tackle the social and economic roots of disadvantage. Drawing on the evidence presented, we argue for swift, evidence‐based action to address the critical state of health in the United States.

## Targeting Risk Factors: Next Step in Closing County‐Level Health Disparities

Our work demonstrates substantial variation in life expectancy across US counties, reflecting deep and persistent health disparities by race, ethnicity, and geography. Building on these findings, we previously showed that behavioral and metabolic risk factors are pivotal, explaining 74% of the variation in county‐level life expectancy, while socioeconomic status and race/ethnicity account for 60%, health care factors for 27%, and all factors combined explain 74%.^31^ While ongoing efforts to address socioeconomic inequalities remain essential, our data highlight that tackling risk factors such as smoking, obesity, physical inactivity, and other health behaviors is critical for narrowing health gaps. Given the wide diversity in county performance, no single solution will fit all communities; instead, we must invest in flexible innovation funds that empower localities to implement, evaluate, and refine strategies tailored to their unique needs. Only interventions with proven effectiveness should be scaled up. Moreover, medical doctors must be engaged not only in direct patient care, but also as partners in prevention, collaborating with population health experts so that hospitals become centers for community well‐being, actively fostering health beyond their walls.

## Growing Burden of Obesity and Metabolic Disease

Among the most urgent health threats facing the United States is the rise of obesity.^14^, ^15^, ^16^, ^17^, ^18^, ^19^, ^20^ Despite decades of warnings, obesity rates have reached historic highs, with profound consequences for morbidity, mortality, and health care costs. Type 2 diabetes prevalence mirrors this trend, now affecting tens of millions and increasingly seen at younger ages.^9^, ^21^, ^32^ These conditions contribute directly to stagnating life expectancy, fueling complications like cardiovascular disease, kidney failure, and amputations. Drivers include poor nutrition, limited access to healthy foods, sedentary lifestyles, and aggressive marketing of unhealthy products. Tackling this epidemic requires a transition from a reactive, acute‐care model to a proactive strategy of national metabolic resilience.

Crucially, metabolic resilience must be integrated into the K‐12 experience through mandatory daily physical activity and high‐quality, minimally processed school meals. Evidence from Chilean school‐based exercise and nutrition programs is instructive, but cautionary: several interventions have failed to reduce, and in some cases coincided with increases in, obesity prevalence, even as scheduled physical activity has been cross‐sectionally associated with better academic performance.^33^, ^34^, ^35^ This suggests that framing movement and nutrition as components of cognitive development and school success may build support even where BMI‐level change lags behind. To support this, investments in the built environment must prioritize physical safety, including protected pedestrian greenways and safe routes to school, ensuring that active living is a safe and accessible default for all communities.

A cornerstone of this transformation must be a fundamental restructuring of the American food environment and subsidies.^36^ The rise in high BMI‐attributable DALYs is a predictable outcome of a system that subsidizes caloric density over nutritional quality and encourages excessive total caloric intake. We propose a bold realignment of federal agricultural policy, shifting subsidies away from corn and soy toward fresh produce to lower their retail cost. Coupled with tiered excise taxes on sugar‐sweetened beverages and a national ban on the marketing of ultra‐processed foods to children, these measures would ensure that the “healthy choice” is no longer a luxury reserved for the most advantaged.

## Confronting Substance Use and Smoking

Substance use disorders, especially involving opioids, stimulants, and alcohol, remain a critical and growing crisis. The opioid epidemic, now compounded by fentanyl and polysubstance use, has overwhelmed communities. Alcohol‐related mortality is also increasing, reversing years of progress. These crises expose the inadequacy of punitive, fragmented approaches to drug policy. Evidence supports investing in comprehensive, evidence‐based prevention and treatment: expanding access to mental health services, integrating harm reduction, and addressing the social determinants that make individuals vulnerable to substance use.^37^, ^38^

Furthermore, while the United States has achieved notable success in reducing tobacco use, this progress is at risk. Emerging products and aggressive industry marketing threaten to stall or reverse declines, especially among youth and marginalized groups. Sustained, evidence‐based tobacco control through taxation, marketing restrictions, and cessation support is essential to preserve gains against one of the nation's deadliest risk factors.^39^

The progress against tobacco use demonstrates what sustained, evidence‐based policy can achieve over time, but the scope and human cost of the substance use crisis demand that we sustain and intensify our efforts on multiple fronts. We therefore recommend the following priorities to further reduce this burden: expand integrated mental health and addiction services as a standard component of primary care rather than a parallel system; invest in economic revitalization and workforce opportunity in the communities most severely affected; and shift performance accountability away from enforcement metrics and toward measurable reductions in overdose mortality and improvements in community well‐being at the county level.

## Targeting Upstream Drivers of Psychological Distress

The escalating burden of mental health disorders in the United States is increasingly driven by environmental and social stressors that bypass traditional clinical interventions. A primary factor is the pervasive impact of digital environments and social media on adolescent neurodevelopment, which has been linked to the precipitous rise in depression and anxiety among youth.^40^, ^41^ Furthermore, structural vulnerabilities, including the prevalence of intimate partner violence, the long‐term sequelae of childhood sexual abuse, and the “normalization” of high‐risk alcohol consumption as a maladaptive coping mechanism create a bidirectional cycle that exacerbates chronic metabolic and cardiovascular diseases.^42^, ^43^, ^44^ Effective public health policy must move beyond clinical management to prioritize primary prevention: regulating digital platforms to protect developmental health, investing in community‐level violence prevention, and addressing the social instability that fuels substance use.

## Integrating Behavioral Health Into Universal Primary Care

While addressing upstream drivers is essential, the US health care system must simultaneously resolve the structural fragmentation that treats mental and physical health as separate entities.^45^ This historical “siloing” undermines comprehensive care, as individuals with untreated mental health conditions face significant barriers to managing chronic physical illnesses like diabetes or cardiovascular disease.^46^, ^47^ We propose a transition toward fully integrated medical‐behavioral models where mental health screenings and evidence‐based interventions are standard components of universal primary care. By treating the “whole person” within a single coordinated system, we can improve adherence to chronic disease protocols and mitigate the risk of clinical escalation, ensuring that the health care system is equipped to handle the complex, multimorbidity profiles of the modern American population.

## Role of Systems and Social Investment

A health system focused almost exclusively on medical intervention, rather than primary prevention, is ill equipped to halt these declines.^48^, ^49^ The United States remains one of the few high‐income countries without universal health insurance, and underinvestment in social programs leaves large segments of the population vulnerable. Comparative studies consistently show that nations with robust social spending and universal coverage achieve better health outcomes at lower per capita cost.

While personal responsibility plays a role, it is shaped by environmental factors. Expecting individuals to make optimal choices in the face of poverty, food insecurity, and targeted marketing of unhealthy products is unrealistic. Government action is essential to create environments where the healthy choice is the easy and affordable choice.

## Building Health Resilience for Future Shocks

Recent acute events have made it clear that national health is tied to our ability to recover from disasters. The devastating wildfires in Hawaii and floods in Texas show how disasters overwhelm health systems. When communities are not robustly healthy to begin with, they take far longer to recover. The COVID‐19 pandemic provided a sobering example: the United States experienced a sharp and sustained drop in life expectancy. These crises underscore the urgent need for a stronger, more equitable health infrastructure. Without proactive investment in preparedness, future disasters will continue to set us back.

## Improving the National Health Data Infrastructure

None of these interventions can be effectively managed without a revolution in our national health data infrastructure. There is an urgent need for timely and open health data systems to maximize public benefits. The era of fragmented, manual, and delayed data reporting must end. We call for a national public health data modernization initiative that leverages AI and small‐area estimation to provide real‐time, neighborhood‐level insights into chronic disease. Only through such a unified, interoperable system can we move toward “Precision Public Health,” holding systems accountable for improving equity based on objective need.

## Role of Education in Health and Economic Vitality

Education is a foundational determinant of both individual and population health.^50^, ^51^, ^52^ Higher educational attainment is consistently linked to longer life expectancy, lower rates of chronic disease, and improved health behaviors. Notably, education enhances economic opportunities, buffers against poverty, and strengthens communities’ resilience to health crises. The economic benefits are substantial: a more educated and healthier workforce drives productivity, innovation, and fiscal sustainability, while also reducing public expenditure on health care and social services.^53^ Conversely, educational disparities exacerbate health inequities, perpetuating cycles of disadvantage across generations. Evidence from both US and international studies demonstrates that investments in early childhood and quality K‐12 education yield profound returns, not only in academic achievement but also in improved physical and mental health, reduced health care costs, and greater social mobility. Therefore, treating education as a fundamental component of health policy is essential to achieving equitable and sustainable improvements in both national well‐being and economic competitiveness.

## Place‐Based Policy and Community Engagement

Our county‐level analyses make clear that the burden of disease and disability is not distributed uniformly across the United States, and that the variation observed within states is often as striking as the variation between them. A one‐size‐fits‐all national policy response is therefore insufficient. Every community holds its own deck of cards, a distinct combination of leading causes of death and disability, dominant risk factors, economic circumstances, and social assets. Effective policy must be built around that local reality rather than imposed upon it. This calls for a fundamental shift in how health policy is designed and governed: away from purely top‐down mandates and toward flexible federal‐state‐local partnerships that empower communities to identify their most pressing health challenges, design contextually appropriate responses, and adapt those responses based on what the local evidence shows is working. A robust literature on community engagement and participatory action research demonstrates that interventions codesigned with the communities they serve are more likely to be adopted, sustained, and effective.^54^, ^55^, ^56^ In practical terms, this requires dedicated local health innovation funds with accountability tied to measurable health outcomes rather than process compliance, allowing communities to test, evaluate, and refine strategies tailored to their unique needs, with the explicit expectation that only those proven effective become candidates for broader scaling.

## Policy Recommendations and Future Directions

To reverse the negative trends identified in this study, the United States must fundamentally realign its national priorities toward primary prevention and health equity. The current model, which treats health care as a market‐based privilege rather than a public utility, is increasingly ill equipped to handle the shift from acute to chronic disabling disease. Expanding access to comprehensive, universal health insurance is a practical and economic imperative. However, as important as it is, coverage alone is insufficient. We propose a tiered policy framework that targets the root causes of disparity through the following four strategic pillars:

1. ***Value Lifelong Education as a Catalyst for Economic and Metabolic Health***. Education is the most potent lever for improving a community's long‐term health. We recommend a national policy that treats education as a longitudinal health intervention:

- *Universal Early Childhood Systems*: The federal government must provide universal day care and pre‐K. This supports parental workforce participation and allows for the early introduction of nutrient‐dense, minimally processed meals. Establishing these dietary patterns before the “adiposity rebound” period is critical to breaking the cycle of metabolic disease in high‐burden counties.
- *Location‐Neutral K‐12 Investment*: National health and economic resilience should not be determined by a student's geographic location. While many states have increased per‐pupil spending in low‐income areas, financial parity has not yet translated into universal instructional quality. Policymakers must move beyond aggregate spending targets to ensure equity in educational content. This requires a national commitment to high‐impact curricula that integrate health literacy, nutrition, and the use of generative AI for personalized, adaptive learning. To achieve this, we must reform the structural reliance on local property taxes, which creates “funding volatility” even in high‐spend districts, and shift toward a stable, federal‐state partnership that scales proven instructional models (such as high‐dosage tutoring) to every school, regardless of neighborhood wealth.^57^, ^58^, ^59^
- *Economic Reintegration Through Higher Education*: As AI and automation shift the labor market, higher education affordability is a necessity. We propose targeted outreach and financial support to bring men back into the higher education system to mitigate the “deaths of despair” and lost human potential observed among young males.
- *Digital Environment Protections*: To protect the neurodevelopmental foundations of learning and mental health, the federal government should implement oversight of social media algorithms. This includes establishing national “digital safety standards” to mitigate the addictive feedback loops identified as upstream drivers of the youth mental health crisis, ensuring that the digital environment supports rather than undermines educational and developmental goals.

2. ***Close the “Convenience Gap” Through Environmental Reform***. Behavioral change is often stimulated by a built environment that favors low levels of physical activity and poor diet. A parent facing “time poverty” will logically choose the fastest, cheapest option for food and exercise. We propose the following:

- *Subsidization of Nutritional Equity*: A shift in agricultural policy to move subsidies away from corn and soy toward specialty crops. This must be paired with “nutritional equity incentives” for local retailers in food deserts to stock affordable healthy meals and scaling mobile “healthy‐food hubs” that integrate clinical screenings with these incentives.
- *Technological Bridging of Food Deserts*: In urban centers, “green delivery incentives” should subsidize fresh produce delivery for low‐income households. In rural regions like Appalachia, we propose federally funded, mobile “healthy‐food hubs” to bring fresh inventory to isolated communities.^60^, ^61^
- *Active Infrastructure Integration*: Federal investments in comprehensive sidewalk networks, intergenerational parks, and pedestrian greenways must lead residents toward healthy‐food access points rather than a gauntlet of fast‐food outlets. These investments must be prioritized to ensure safe, walkable access to exercise for all ages.

3. ***Target Medical Escalation: Normalizing Chronic Obesity Management***. We must admit that behavioral advice alone has failed. Obesity must be reclassified as a chronic biological condition requiring long‐term clinical management:

- *Universal Coverage for Pharmacotherapy*: A federal mandate to cover next‐generation anti‐obesity medications, such as GLP‐1 receptor agonists, through Medicare and Medicaid. These must be removed from the “lifestyle” category and integrated into the standard of care.
- *Federal Research and Development Investment and Market Modernization*: We call for a federal strategic initiative to accelerate the development and domestic production of next‐generation metabolic regulators (such as GLP‐1/GIP agonists) with improved side effect profiles. A primary goal must be the democratization of the peptide market to achieve price parity with daily statins.^62^ Current market distortions where the final prescription price represents a massive markup over the negligible cost of the underlying peptide create profound and unnecessary health disparities. To bypass these barriers, we propose regulatory reforms that expand safe, high‐quality compounding and domestic manufacturing pathways. By modernizing the supply chain and generic entry, we ensure that metabolic resilience is no longer a premium service for the wealthy but a standard, affordable component of universal primary care.
- *Accountability Through Precision Public Health*: An aggressive risk‐monitoring system should be employed to evaluate the effectiveness of these medications at the neighborhood level, holding systems accountable for reductions in cardiovascular and renal risk.

4. ***Address the Drug Use Epidemic***. The data from this analysis highlight the scale of the drug use epidemic and its contribution to the overall burden of disease in the United States. Drug use disorders have risen from the 26th cause of DALYs in 1990 to the leading cause by 2023, a trend that warrants targeted policy attention alongside the metabolic and behavioral interventions described earlier. Addressing this epidemic requires treating drug use disorders as chronic conditions within an integrated care framework, rather than as primarily criminal justice matters:

- *Strengthening of Insurance Parity for Substance Use Disorder Treatment*: The Paul Wellstone and Pete Domenici Mental Health Parity and Addiction Equity Act of 2008 established that insurance coverage for substance use disorders should be no more restrictive than coverage for other medical conditions.^63^ Consistent enforcement of this requirement, particularly with respect to utilization management practices and network adequacy, would help ensure that evidence‐based treatment is accessible to those who need it, including in the communities identified in this analysis as carrying the greatest burden.
- *Expansion of Access to Medications for Opioid Use Disorder*: Buprenorphine, methadone, and naltrexone are among the most effective interventions available for opioid use disorder, yet access has historically been constrained by administrative barriers, geographic maldistribution of providers, and pharmacy‐level restrictions. The elimination of the US Drug Enforcement Administration X‐Waiver requirement in 2023 reduced one important regulatory hurdle^64^; however, significant gaps in access persist, particularly in rural and underserved areas. Sustained investment in colocating medications for opioid use disorder within primary care and safety‐net settings would help bring treatment to the populations most affected.
- *Integration With Broader Social and Behavioral Health Strategies*: As documented throughout our research, the drug use epidemic intersects with the socioeconomic disadvantage, educational disinvestment, and mental health burden that drive inequity across the United States. Embedding substance use disorder screening and treatment within primary care and community health settings, consistent with integrated care models supported by the evidence base, would complement upstream investments in education and environment as described in pillars 1 and 2, and help address the co‐occurring conditions that sustain this burden over time.

## Limitations

As with any large‐scale epidemiological analysis, several limitations must be acknowledged. The strengths and weaknesses of the GBD methodology have been widely discussed, and many apply to this US‐focused assessment. A major constraint is the variability and completeness of available data across states and over time. Accurately capturing dietary intake at the state level, for example, often requires reliance on commercial sales data and indirect estimation methods, which may introduce imprecision. In some instances, underlying datasets, whether for risk factors or disease outcomes, are inconsistent, affecting estimate accuracy.

Distinguishing true changes in disease rates from measurement error is another challenge. Although GBD employs rigorous statistical adjustments for nonstandard definitions and known biases, sparse state‐level data can limit reliability. Hospital admission data further complicate analysis: administrative claims databases may not fully represent all populations or care settings, and differences in coding practices and management across states add uncertainty.

This analysis scope was limited to behavioral, metabolic, and environmental/occupational risks, and did not directly assess broader social determinants such as housing instability, exposure to discrimination, neighborhood safety, and transportation, all of which significantly shape health outcomes.

Despite these constraints, the study yields important and actionable insights into US health patterns and disparities. Continued improvement in data collection, analytical methods, and computational capacity will be essential for refining future estimates and expanding our understanding of disease burden. Addressing these limitations will strengthen the evidence base and support more effective health policy and resource allocation.

## Conclusion

The United States stands at a critical juncture in its public health history. As our analysis of the Ten Americas demonstrates, the current national health landscape is defined not by a unified trajectory of progress but by a widening chasm of inequity where a citizen's race, ethnicity, and geography determine their very capacity to survive. The transition from fatal to disabling disease, fueled by the relentless surge in obesity and metabolic risk, is a systemic failure that cannot be cured by the medical system alone. To reverse these trends, we must embrace a multidimensional policy agenda that prioritizes early childhood education as a health intervention, supports behavioral modification through environmental change, and ensures equitable access to lifesaving pharmacotherapy for those who need it most. We must also recognize that none of these efforts can succeed without a modernized, transparent data infrastructure to guide our actions. Ultimately, the health of the nation is not merely a clinical metric but a reflection of our collective commitment to human potential. The time for incremental change has passed; we must now choose to build a future where every American can lead a long, healthy, and fulfilling life.

## Funding/Support

Gates Foundation

Intramural Research Program, National Institute on Minority Health and Health Disparities, US National Institutes of Health (contract #75N94023C00004)

The funder(s) had no role in the design of the study; the collection, analysis, or interpretation of the data; or the writing of the manuscript and decision to submit it for publication.

## Conflict of Interest Disclosures

All authors have no conflict
